# Influence of cyclodextrin on the solubility and the polymerization of (meth)acrylated Triton^®^ X-100

**DOI:** 10.3762/bjoc.8.245

**Published:** 2012-12-13

**Authors:** Melanie Kemnitz, Helmut Ritter

**Affiliations:** 1 Institute of Organic Chemistry and Macromolecular Chemistry II, Heinrich-Heine-University, Universitätsstraße 1, 40255 Düsseldorf (Germany)

**Keywords:** (meth)acrylated Triton^®^, randomly methylated β-cyclodextrin (RAMEB-CD), rheology, Triton^®^ X-100 (poly(ethylene glycol)*tert*-octylphenyl ether)

## Abstract

Triton^®^ X-100 (poly(ethylene glycol) *tert*-octylphenyl ether) was (meth)acrylated and polymerized in the absence and presence of randomly methylated β-cyclodextrin (RAMEB-CD). Triton^®^-polymers that were polymerized with RAMEB-CD in water were compared with polymers that were synthesized in organic solvents after the addition of RAMEB-CD. The polymers were characterized by ^1^H NMR and FTIR spectroscopy, matrix-assisted laser desorption ionization mass spectrometry (MALDI-TOF MS), dynamic light scattering (DLS), gel-permeation chromatography (GPC) and turbidity measurements. Additionally, the viscosity change of the methacrylic homopolymer with RAMEB-CD was evaluated.

## Introduction

Triton^®^ X-100 (**1**) is a macromolecular, nonionic surfactant with an average number of ethylene oxide units of 9.5. The *tert*-octylphenyl group is a hydrophobic moiety whereas the poly(ethylene glycol) substituent is hydrophilic. Because of this amphiphilic character, Triton^®^ X-100 can be described as a short AB block co-oligomer [1]. Long amphiphiles are relatively flexible and provide a lower critical micelle concentration (CMC) [2]. The CMC of Triton^®^ X-100 is 0.22 mM [3]. Accordingly, below this concentration isolated molecules can be found; above this concentration micelles are formed.

Equilibrium constants of β-CD and Triton^®^ X-100 complexes listed in the literature span a range of 145–171000 M^−1^. Different methods, e.g., surface tension, isothermal titration calorimetric, and fluorescence studies, were used to determine the equilibrium constant [4–12]. The results also differ in the stoichiometry of the formed complexes [4,7,9–11]. Host/guest ratios of 1:1 [4,7], 2:1 [9] and the coexistence of 1:1 and 2:1 [10–11] complexes of β-CD and Triton^®^ X-100 are reported in literature. In our previous paper, the coexistence of a 1:1 and 2:1 complex with an extraordinary high (*K*_1_ = 1.71 × 10^5^ M^−1^) and a lower equilibrium constant (*K*_2_ = 260 M^−1^) was described [12]. Due to the fact that the *tert*-octyl group represents the preferred binding site of the molecule it can be assumed that complexation takes place at this position first. In excess of β-CD an additional complexation of the phenyl ring occurs.

The synthesis and polymerization of methacrylic Triton^®^ has already been described in the literature [2]. It was found that the reactivity of the methacrylic double bond was not affected by the length of the poly(ethylene oxide) side chain [13].

In the present work, we depict the homopolymerization and characterization of methacrylic and acrylic Triton^®^ in the absence and presence of RAMEB-CD for the first time. The dissociation of the polymer-CD-complex was monitored by viscosity measurements.

## Results and Discussion

### Complexation study of Triton^®^ X-100 with RAMEB-CD

The complexation behavior of Triton^®^ X-100 (**1**) with RAMEB-CD was investigated by means of an ITC experiment (Supporting Information File 1, Figure S1). Assuming the sequential-binding model with two binding sites, values of *K*_1_ = 6.08 × 10^4^ M^−1^ and *K*_2_ = 445 M^−1^ were obtained, which are extremely high compared to the other CD-complexes which show *K*-values rarely higher than 5 × 10^4^ M^−1^ [14].

**Figure 1:**
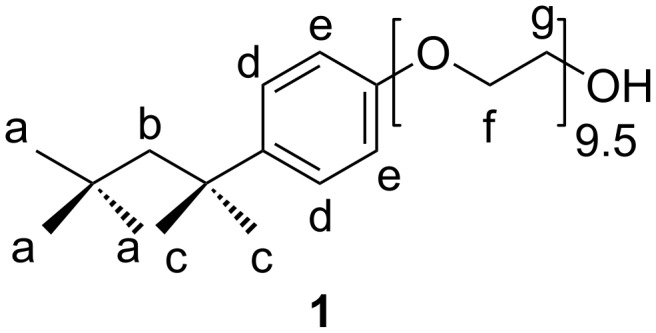
Chemical structure of Triton^®^ X-100 (**1**).

^1^H NMR spectroscopy experiments indicate that Triton^®^ is complexed at its hydrophobic *tert*-octyl-moiety as expected. The ^1^H NMR signals of the aliphatic protons **a** and **c** at 0.66 and 1.24 ppm (Figure 1) shifted strongly after the addition of a small amount of RAMEB-CD. With increasing amount of RAMEB-CD, the signals of the aromatic protons **d** and **e** also shifted from 7.16 and 6.78 ppm to 7.34 and 6.93 ppm. Assumingly, the hydrophobic *tert*-octyl group is complexed first; secondly the aryl group is complexed by RAMEB-CD [12].

To evaluate the size of Triton^®^ X-100 (**1**) and its supramolecular complexes with CD in water, DLS measurements were carried out. The number-average hydrodynamic diameter (*d*_h_) increased from 0.7 nm at concentrations lower than 0.22 mM up to 106 nm indicating the formation of micelles above the CMC. The formation of micelles was inhibited by the addition of RAMEB-CD to the Triton^®^ solution, because the hydrophobic component of **1** slips into the cavity of RAMEB-CD. The addition of 1 equiv of RAMEB-CD to **1** exhibited a hydrodynamic diameter of 1.4 nm and the diameter of the 1:2 complex was determined to 2.1 nm. The different hydrodynamic diameters of the complexes indicate that different complexes are formed.

**Figure 2:**
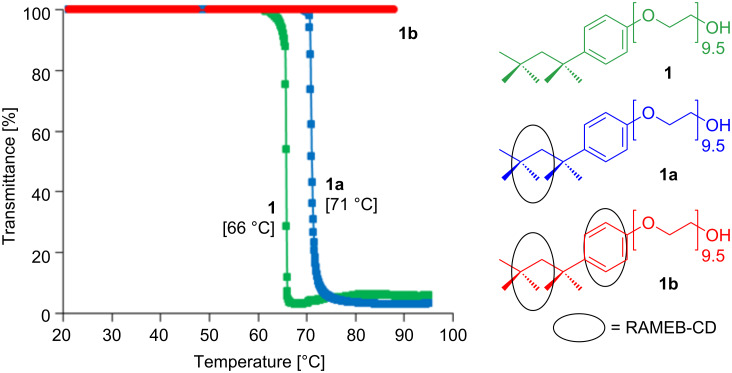
Solubility in water of 0.2 wt % Triton^®^ X-100 (**1**) and its different assumed complexes with RAMEB-CD (**1a** and **1b**) according to the transparence measurement.

Triton^®^ X-100 (**1**) as a macromolecular surfactant becomes insoluble above 66 °C due to a typical LCST effect. By addition of 1 equiv of RAMEB-CD (**1a**) the cloud point is shifted from 66 to 71 °C. This can be attributed to the increasing hydrophilicity. Figure 2 shows the changes of the transmittance as a function of the temperature. After the addition of a second equiv of RAMEB-CD (**1b**) no LCST behavior could be observed over the whole temperature range from 5 to 95 °C. This was due to the much more hydrophilic character of the complex, which is a result of coverage of the hydrophobic part of **1**.

### Complexation of the (meth)acrylic monomer derived from Triton^®^ (**2** and **3**) with RAMEB-CD

**Scheme 1:**
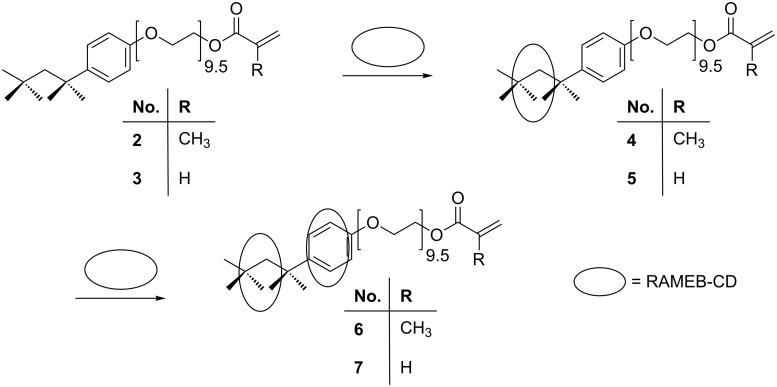
Idealized reaction of the complexation of the (meth)acrylic monomer derived from Triton^®^ (**2** and **3**) with one and 2 equiv of RAMEB-CD.

The reaction of Triton^®^ X-100 (**1**) with methacryloyl chloride and acryloyl chloride gave the corresponding methacrylic ester **2** [2] and acrylic ester **3**, respectively. Both monomers **2** and **3** were insoluble in water, but readily formed water-soluble host–guest complexes with RAMEB-CD (**4** to **7**) within a few minutes (Scheme 1).

In the solid state, the complex formation could be proved by FTIR, as the ether-band shifted from 1102 to 1033 cm^−1^. By using 2D-ROESY NMR, the interactions between the inner protons H-3 and H-5 of 2,6-dimethyl-β-cyclodextrin (DIMEB-CD) and the protons of the tert-octyl and aryl group of **3** were analyzed (Supporting Information File 1, Figure S2). DIMEB-CD was used with the intention to gain information on the orientation of the RAMEB-CD molecules on Triton^®^. However, after the complexation, the signals of the inner protons were superimposed and no conclusions about the orientation of the CD ring could be drawn. An interaction between CD and the (meth)acrylic group could not be detected.

In the course of DLS experiments with dimethylformamide (DMF) as solvent a hydrodynamic diameter of 1.4 nm for pure RAMEB-CD and 1.2 nm for **3** was found. For each complexed RAMEB-CD the diameter increased by about 0.6 nm. Therefore, it can be concluded that RAMEB-CD forms stable complexes with the modified (**2** and **3**) and unmodified Triton^®^ X-100 (**1**) even in DMF as solvent (Table 1).

**Table 1:** Comparison of the hydrodynamic diameters and the LCSTs of **1**, **2** and **3** and their complexes with 1 and 2 equiv of RAMEB-CD.

compound	hydrodynamic diameter (nm) in DMF			LCST (°C) in water		
	without RAMEB-CD	with RAMEB-CD		without RAMEB-CD	with RAMEB-CD	
		1 equiv	2 equiv		1 equiv	2 equiv

**1**	106	1.4	2.1	66	71	not measurable
**2**	1.3	1.8	2.5	insoluble	10	27
**3**	1.2	1.8	2.5	insoluble	18	63

Furthermore, the water-insoluble monomers **2** and **3** can be transferred into the aqueous phase by addition of RAMEB-CD. Above 27 °C the 2:1 complex of RAMEB-CD with the methacrylic monomer **2** precipitates as a result of slipping off the RAMEB-CD. If the solution is cooled down the complex is reformed and the dispersion became completely transparent again. With increasing concentration of RAMEB-CD and decreasing hydrophobicity of the modified monomers **2** and **3** the LCST increases significantly (Table 1).

### Homopolymerization of the uncomplexed (meth)acrylic monomers **2** and **3** in DMF

**Scheme 2:**
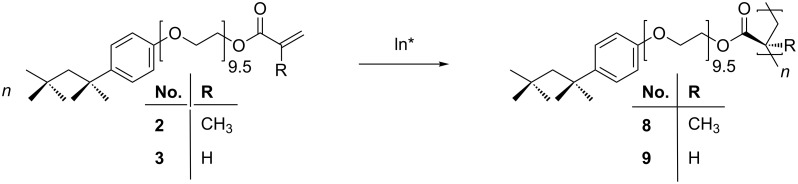
Homopolymerization of the uncomplexed monomers **2** and **3** to the polymers **8** and **9** in DMF with AIBN as initiator.

The macromonomers **2** and **3** respectively were homopolymerized in DMF with 2,2′-azobis(2-methylpropionitrile) (AIBN) as initiator (Scheme 2). The glass-transition temperature (*T*_g_) of the methacrylic polymer **8** was found to be −40 °C, which is similar to the value that can be found in the literature [2]. Due to higher flexibility of the new acrylic polymer **9**, a lower *T*_g_ of −50 °C was detected.

According to DLS measurements, the addition of 1 equiv of RAMEB-CD to the dissolved polymer does not significantly influence the hydrodynamic diameter of 10.4 nm (**8**) and 11.7 nm (**9**) (+1 nm) in DMF. In contrast, the complexation with 2 equiv of RAMEB-CD induces a remarkable shift in *d*_h_ from 10.4 up to 18.4 nm (Table 2). This observation indicates that a total complexation of the hydrophobic component is required to prevent the intermolecular aggregation of the coil, which results in a higher rigidity of the polymer chain.

**Table 2:** The hydrodynamic diameters and molecular weights of the polymers **8** and **9** and their complexes with RAMEB-CD in DMF.

compound	hydrodynamic diameter (nm) in DMF			GPC (DMF)	
	without RAMEB-CD	with RAMEB-CD		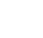	*D*
		1 equiv	2 equiv		

**8**	10.4	11.4	18.4	80900	4.9
**9**	11.7	12.5	16.3	46800	6.5

The addition of 2 equiv of RAMEB-CD to **8** leads to a water soluble polymer with a cloud point of 11 °C. For the single and double RAMEB-CD-complexed acrylic polymer **9** no cloud points could be observed over the whole temperature range, probably because the complexes are too stable in water.

### Homopolymerization of RAMEB-CD complexed (meth)acrylic monomer derived from Triton^®^ (**4** to **7**) in water

**Scheme 3:**
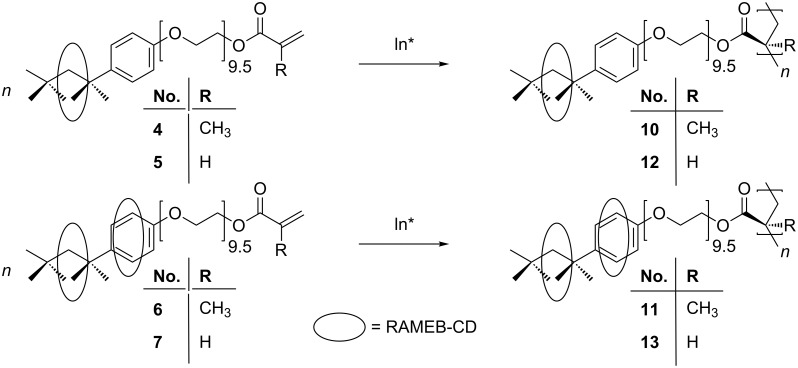
Polymerization of the RAMEB-CD complexed monomers **4**, **5**, **6** and **7** to the homopolymers **10**, **11**, **12** and **13** in water with VA-044 as initiator.

A solution polymerization in water was possible after the complexation of (meth)acrylated Triton^®^ X-100 (**2** and **3**) with RAMEB-CD (Scheme 3). The polymers **10** and **12** complexed with 1 equiv of RAMEB-CD precipitated during the polymerization, whereas the double complexed polymers **11** and **13** did not precipitate, as a consequence of the stronger host–guest interactions.

The successful polymerization of **10**–**13** was demonstrated, e.g., by ^1^H NMR spectroscopy. The signals of the olefinic protons between 5.5 and 6.2 ppm vanished; whereas the signals of the polymer backbone at 0.67 ppm were clearly visible. As expected, the signals of the protons of the *tert*-octyl group were broader after the polymerization. The 2D-ROESY spectrum indicates that the complex was still intact after the polymerization (Supporting Information File 1, Figure S3).

DSC measurements of the dried samples indicate a high influence of RAMEB-CD. The *T*_g_ value was found to be about 52 °C for the single complexed acrylic polymer **12** and about 85 °C for the double complexed acrylic polymer **13**. Compared to this a *T*_g_ value of −50 °C was found for the model polymer **9**. Obviously, RAMEB-CD reduces the mobility of the system, as suggested above. A similar behavior was observed for the single complexed methacrylic polymer **10**. The hydrodynamic diameters of the complexed, modified monomers are between 1.4 and 2.5 nm. For the polymers **10**–**13**, which were first complexed with RAMEB-CD and then polymerized in water, and the polymers **8** and **9**, which were homopolymerized in DMF and then complexed with RAMEB-CD, similar hydrodynamic diameters were observed (Supporting Information File 1, Table S1). To determine the molecular weight of the polymers RAMEB-CD had to be removed first. This was achieved by a dialysis at 45 °C (Table 3). The molecular weights of the polymers **11** and **13** derived from the double-complexed monomers **6** and **7** are higher than the molecular weights of the polymers **10** and **12,** which were prepared from the single-complexed monomers **4** and **5**. This may be a result of the increasing solubility of the monomers **2** and **3** if CD is added in molar excess.

**Table 3:** Molecular weights of the polymers **10**–**13** after dialysis at 45 °C.

compound	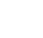	*D*

**10**	119200	7.3
**11**	175300	3.4
**12**	54700	3.3
**13**	68400	3.2

### Temperature-dependent behavior of the RAMEB-CD-double-complexed methacrylic homopolymer (**8**)

**Figure 3:**
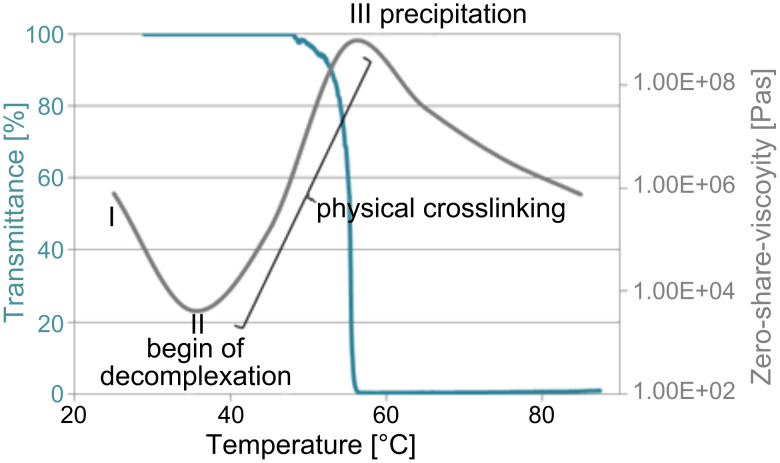
Transmittance [%] and zero-shear-viscosity [Pas] as a function of temperature for a 50 wt % solution of polymer **8**. (**I** → **II**: Viscosity decreases with higher temperature; **II** → **III**: Gradual decomplexation of the polymer, RAMEB-CD molecules slip off from the polymer side chain, aggregates are formed; **III**: Precipitation of the polymer).

The rheological experiments were performed in a temperature range of 25 to 85 °C for constant shear rates. Below 30 °C the viscosity of an aqueous polymer solution decreases with increasing temperature (Figure 3, I). Surprisingly, above 30 °C the viscosity increases (Figure 3, II). Probably this result is caused by a gradual decomplexation of the polymer. Some RAMEB-CD molecules slip off from the polymer side chain and aggregates are formed. The agglomeration causes an increase in the viscosity, with its maximum at about 60 °C. Above this temperature, the polymer precipitates (Figure 3, III). Similar curves were determined for different shear rates and the solution showed shear-thinning behavior. The results found by rheometer were confirmed by DLS and turbidity measurements. The cloud point was around 56 °C. Figure 3 shows the transmittance and the zero-shear-viscosity as a function of the temperature in a range of 25 and 85 °C for a 50 wt % aqueous solution of **8**. Approaching the LCST, the hydrodynamic diameters increase (16 nm at 25 °C, 29 nm at 30 °C, 62 nm at 35 °C, 77 nm at 40 °C and 95 nm at 45 °C). The results of hydrodynamic diameters and the transmittance support the described explanation for the viscosity curve on the rheometer.

## Conclusion

It was possible to polymerize (meth)acrylated Triton^®^ as a stable complex with RAMEB-CD in water (**10** to **13**). Even after the polymerization, the complex with RAMEB-CD still existed. Water is an environmentally friendly and favorable solvent. The molecular weights, LCSTs and hydrodynamic diameters of the resulting polymers prepared in water are similar to those of the uncomplexed model polymers, which were synthesized in DMF (**8** and **9**). As an important result, it was possible to follow the decomplexation of double-complexed polymer **8** above 30 °C by DLS, turbidity measurements and also by viscosity changes.

## Experimental

### Materials

Triton^®^ X-100 was purchased from Acros Organics and was used as received. RAMEB-CD was bought from the Wacker Chemie AG, and DIMEB-CD was bought from Acros Organics. They were dried in a microwave drying system SAM 255 (CEM). Methacryloyl chloride (Fluka), acryloyl chloride (Fluka), triethylamine (Acros Organics), AIBN (Sigma-Aldrich), VA-044 (Wako Chemicals) and DMF (VWR) were used as received.

### Measurements

Isothermal titration calorimetry experiments were performed on a VP-ITC MicroCalorimeter from MicroCal, Northampton MA, and were controlled by MicroCal VP viewer 2000 ITC software. Experiments were conducted at 25 °C. A 2.5 mM solution of RAMEB-CD was titrated against a 0.2 mM solution of Triton^®^ X-100. Titration was carried out by 30 injections of 5 × 1 μL, 5 × 2 μL, 5 × 5 μL, 15 × 15 μL with a spacing time of 180 s. The stirring speed was set to 300 min^−1^. In a separate experiment the heat of dilution was measured and was subtracted from the binding isotherm. The results were calculated with Origin 7.0 and the MicroCal LLC ITC software.

2D-ROESY and ^1^H NMR measurements were performed on a Bruker AVIII-300 spectrometer at 300.13 MHz. The δ scale relative to tetramethylsilan was calibrated to the solvent value δ 7.26 ppm for CDCl_3_ and δ 4.79 ppm for D_2_O.

Infrared (IR) spectra were determined on a Nicolet 6700 FTIR spectrometer equipped with an ATR unit at rt. The measurements were performed in the range of 4000–300 cm^−1^.

DLS data were recorded in backscattering mode on a Malvern Zetasizer Nano ZS ZEN 3600 at a temperature of 20 °C with a laser wavelength of 633 nm and a detection angle of 173°. Measured solutions contained 5 mg/mL substance in DMF and were performed in a glass cuvette with a layer thickness of 1 cm. Each experiment was performed at least ten times to obtain statistical information and the number-averaged diameters were used for characterization.

For turbidity measurements a TP1 turbidity photometer within a temperature range of 5 to 95 °C was used. Measured solutions contained 20 mg/mL substance in water. During continuous stirring the transparency of the sample was determined. The values of the LCST were taken as the temperature where the transmission decreases by 50%.

GPC measurements were performed by using a Viscotek GPCmax VE2001 system. The system has a column set with one Viscotek TSK guard column HHR-H 5.0 mm (ID) × 8 cm (L) and two Viscotek TSK GMHHR-M 7.8 mm (ID) × 30 cm (L) columns. The columns were constantly heated at a temperature of 60 °C. DMF (0.1 M LiCl) was used as eluent at a flow rate of 1 mL/min. For detection a Viscotek VE 3500 RI detector was used. The system was calibrated with polystyrene standards within a molecular range from 575 to 3,114,000 g/mol.

On a Mettler Toledo DSC 822 instrument the DSC measurements were determined in a range of −50 to 200 °C at the heating rate of 10 °C/min. The *T*_g_ values were estimated as the average of three measurements by using the midpoint method.

Viscosities were measured with a rotational viscometer model Haake MARS II rheometer equipped with plate–plate (PP35Ti) and cone–plate (DC60/2°) configuration. A temperature control system DC30/K10 from Thermo scientific was used to ensure constant temperatures with deviations of 1 °C. Measured solutions contained 50 wt % aqueous solution of the polymer. To determine the viscosity a temperature range of 25 to 85 °C (∆*T* = 5 °C) for constant shear rates (10, 20, 100 and 200 s^−1^) was applied. For the zero-shear viscosity, different shear rates (10^−5^–0.1 s^−1^) at constant temperatures from 25 to 85 °C (∆ = 5 °C) were used.

### Syntheses

#### Monomer syntheses

The methacrylic derivate of Triton^®^ X-100 (**2**) was synthesized in a similar way as already described by San Román et al. [2]. To a solution of 9 g (14 mmol) Triton^®^ X-100 (**1**) in methylene chloride with a 20% excess triethylamine (1.7 g, 16.7 mmol), a solution of 20% excess methacryloyl chloride (1.75 g, 16.7 mmol) was added dropwise at 0 °C under stirring. After being stirred for 24 h at rt, the solution was washed with aqueous NaOH (5 wt %) several times. The solvent was removed under reduced pressure. The acrylic derivate of Triton^®^ X-100 (**3**) was synthesized in the same way; instead of methacryloyl chloride, acryloyl chloride (1.5 g, 16.5 mmol) was used.

#### Methacrylic derivate of Triton^®^ X-100 (**2**)

Yield: 82%; FTIR (diamond, cm^−1^): ν 2949/2868 (-CH_3_, -CH_2_-), 1718 (-COOR), 1638 (-C=C-), 1610/1511 (-aryl), 1454, 1365, 1349, 1319, 1296, 1245 (aryl-C-O-C), 1103 (-C-O-C-), 1039, 942 (R_2_C=CH_2_), 829/732 (1,4-disubstitute aromatic), 683, 659, 589, 522; ^1^H NMR (300 MHz, CDCl_3_) δ 7.25 (dd, ^3^*J* = 8.83 Hz, ^4^*J* = 2.18 Hz, 2H, aryl-CH), 6.82 (dd, ^3^*J* = 8.83 Hz, ^4^*J* = 2.18, 2H, aryl-CH), 6.13 (m, 1H, -CH_3_), 5.57 (m, 1H, =CH_2_), 4.29 (t, ^3^*J* = 4.86 Hz, 2H, -CH_2_-), 4.10 (t, ^3^*J* = 4.86 Hz, 2H, -CH_2_-), 3.64 (s, polyethoxy, 37H), 1.94 (t, ^4^*J* = 1.13 Hz, ^4^*J* = 1.33 Hz, 3H, =CH_2_), 1.69 (s, 2H, -CH_2_-), 1.33 (s, 6H, -(CH_3_)_2_), 0.70 (s, 9H, -(CH_3_)_3_); MALDI-TOF (CHCl_3_, *n* = number of ethoxy groups) *m*/*z*: 561 (*n* = 6), 605 (*n* = 7), 649 (*n* = 8), 693 (*n* = 9), 737 (*n* = 10), 781 (*n* = 11), 825 (*n* = 12), 869 (*n* = 13), 913 (*n* = 14), 957 (*n* = 15), 1001 (*n* = 16), 1045 (*n* = 17), 1089 (*n* = 18).

#### Acrylic derivate of Triton^®^ X-100 (**3**)

Yield: 97%; FTIR (diamond, cm^−1^): ν 2952/2868 (-CH_3_, -CH_2_-), 1724 (-COOR), 1636 (-C=C-), 1610/1511 (-aryl), 1455, 1407, 1365, 1350, 1319, 1295, 1245 (aryl-C-O-C), 1188, 1102 (-C-O-C-), 1066, 942 (R_2_C=CH_2_), 829/732 (1,4-disubstitute aromatic), 683, 659, 589, 522; ^1^H NMR (300 MHz, CDCl_3_) δ 7.24 (dd, ^3^*J* = 8.83 Hz, ^4^*J* = 2.16, 2H, aryl-CH), 6.80 (dd, ^3^*J* = 8.87 Hz, ^4^*J* = 2.16, 2H, aryl-CH), 6.41 (dd, ^2^*J* = 1.50 Hz, ^3^*J* = 17.39 Hz, 1H, -H), 6.14 (dd, ^3^*J* = 17.39 Hz, ^3^*J* = 10.44 Hz, 1H, =CH_2_), 5.82 (dd, ^2^*J* = 1.50 Hz, ^3^*J* = 10.44 Hz, 1H, =CH_2_), 4.30 (t, ^3^*J* = 4.86 Hz, 2H, -CH_2_-), 4.10 (t, ^3^*J* = 4.93 Hz, 2H, -CH_2_-), 3.63 (s, 37H, polyethoxy), 1.68 (s, 2H, -CH_2_-), 1.32 (s, 6H, -(CH_3_)_2_), 0.69 (s, 9H, -(CH_3_)_3_); MALDI-TOF (CHCl_3_, *n* = number of ethoxy groups) *m*/*z*: 547 (*n* = 6), 591 (*n* = 7), 635 (*n* = 8), 679 (*n* = 9), 723 (*n* = 10), 767 (*n* = 11), 811 (*n* = 12), 855 (*n* = 13), 899 (*n* = 14), 943 (*n* = 15), 987 (*n* = 16), 1031 (*n* = 17), 1075 (*n* = 18), 1119 (*n* = 19).

#### Homopolymerization in the absence of RAMEB-CD in DMF

The (meth)acrylic derivate of Triton^®^ X-100 (**2** = 10 g, 13.99 mmol; **3** = 10 g, 14.27 mmol) was homopolymerized at 70 °C in a thermostatic bath, with 1 mol % AIBN (0.14 mmol) as radical initiator and 50 wt % dimethylformamide as solvent for 24 h. The polymerization reactions were carried out under an oxygen-free N_2_ atmosphere. The polymer was precipitated in methanol, filtered off and vacuum dried at room temperature until a constant weight was reached.

#### Methacrylic polymer **8**

Yield: 65%; FTIR (diamond, cm^−1^): ν 2948/2868 (-CH_3_, -CH_2_-), 1728 (-COOR), 1609/1512 (-aryl), 1455, 1394, 1364, 1349, 1325, 1294, 1245 (aryl-C-O-C), 1149, 1101 (-C-O-C-), 1038, 947, 829/748 (1,4-disubstitute aromatic), 732, 683, 588, 554, 521; GPC (DMF): 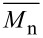 = 80,900, 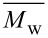 = 396,700, *D* = 4.9.

#### Acrylic polymer **9**

Yield: 74%; FTIR (diamond, cm^−1^): ν 2946/2868 (-CH_3_, -CH_2_-), 1733 (-COOR), 1610/1512 (-aryl), 1455, 1391, 1365, 1349, 1294 (aryl-C-O-C), 1245, 1185, 1103 (-C-O-C-), 1039, 948, 829 (1,4-disubstitute aromatic), 683, 588, 522; ^1^H NMR (300 MHz, CDCl_3_) δ 7.24 (d, ^3^*J* = 8.76 Hz, 2H, aryl-CH), 6.80 (d, ^3^*J* = 8.76 Hz, 2H, aryl-CH), 4.09 (t, ^3^*J* = 4.87 Hz, 2H, -CH_2_-), 3.83 (t, ^3^*J* = 4.87 Hz, 2H, -CH_2_-), 3.63 (s, 37H, polyethoxy), 2.21 (d, ^3^*J* = 8.58 Hz, 2H, backbones), 1.68 (s, 2H, -CH_2_-), 1.32 (s, 6H, -(CH_3_)_2_), 0.70 (s, 9H, -(CH_3_)_2_); GPC (DMF): 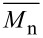
= 46,800, 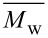 = 303,800, *D* = 6.5.

#### Homopolymerization in the presence of RAMEB-CD in water

The (meth)acrylic derivate of Triton^®^ X-100 (**2** = 1 g, 1.4 mmol; **3** = 1 g, 1.43 mmol) and 1 or 2 equiv of RAMEB-CD were dissolved in water (20 wt % with reference to the monomer) and stirred until the solution became clear. The complexed monomers were homopolymerized at 40 °C in a thermostatic bath, with 1 mol % VA-044 (for **2** 4.6 mg, 0.014 mmol; for **3** 4.8 mg, 0.015 mmol) as radical initiator for 24 h. The polymerization reactions were carried out under an oxygen-free N_2_ atmosphere. The polymers were purified by dialysis for two days.

#### Methacrylic polymer with 1 equiv of RAMEB-CD (**10**) and 2 equiv of RAMEB-CD (**11**)

Yield **10**: 37%; yield **11**: 89%; FTIR (diamond, cm^−1^): ν 3404 (-OH), 2924/2886/2832 (-CH_3_, -CH_2_-, -OCH_3_), 1728 (-COOR), 1610/1512 (-aryl), 1454, 1407, 1364, 1325, 1299, 1246 (aryl-C-O-C), 1188, 1147, 1083, 1034 (-C-O-C-), 965, 858/757 (1,4-disubstitute aromatic), 701, 571; ^1^H NMR (300 MHz, D_2_O, **10** and **11** have identical chemical shifts, but the integral of RAMEB-CD for **11** is a 1.8 times bigger than for **10**) δ 7.35 (d, 2H, ^3^*J* = 8.43 Hz, aryl-CH), 6.95 (d, ^3^* J* = 8.43 Hz, 2H, aryl-CH), 5.20 (2s, RAMEB-CD), 4.34 (m, 2H, -CH_2_-), 4.23 (m, 2H, -CH_2_-), 3.88 (s, RAMEB-CD), 3.81 (s, RAMEB-CD), 3.69 (s, 37H, polyethoxy), 3.67 (s, RAMEB-CD), 3.58 (s, CH_3_- RAMEB-CD), 3.54 (s, RAMEB-CD), 3.40 (s, CH_3_- RAMEB-CD), 2.07 (s, 1H, -CH_3_), 1.77 (s, 2H, -CH_2_-), 1.68 (s, 2H, backbones), 1.51 (s, 6H, -(CH_3_)_2_), 0.85 (s, 9H, -(CH_3_)_2_); GPC (**10**, DMF): 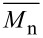 = 119,200, 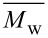 = 876,100, *D* = 7.3; GPC (**11**, DMF): 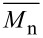 = 175,300, 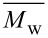 = 593,500, *D* = 3.4.

#### Acrylic polymer with 1 equiv of RAMEB-CD (**12**) and 2 equiv of RAMEB-CD (**13**)

Yield **12**: 45%; yield **13**: 84%; FTIR (diamond, cm^−1^): ν 3405 (-OH), 2924/2896/2832 (-CH_3_, -CH_2_, -OCH_3_), 1731 (-COOR), 1610/1512 (-aryl), 1453, 1404, 1364, 1325, 1299, 1246 (aryl-C-O-C), 1189, 1150, 1083, 1033 (-C-O-C-), 1002, 965, 857/758 (1,4-disubstitute aromatic), 701, 571; ^1^H NMR (300 MHz, D_2_O, **12** and **13** have identical chemical shifts, but the integral of RAMEB-CD for **11** is 1.8 times larger than for **10**) δ 7.35 (d, ^3^*J* = 8.73 Hz, 2H, aryl-CH), 6.94 (d, ^3^*J* = 8.73 Hz, 2H, aryl-CH), 5.27 (2s, RAMEB-CD), 4.34 (m, 2H, -CH_2_-), 4.23 (m, 2H, -CH_2_-), 3.87 (s, RAMEB-CD), 3.70 (s, RAMEB-CD), 3.67 (s, 37H, polyethoxy), 3.67 (s, RAMEB-CD), 3.59 (s, CH_3_- RAMEB-CD), 3.53 (s, RAMEB-CD), 3.40 (s, CH_3_- RAMEB-CD), 1.77 (s, 2H, -CH_2_-), 1.68 (s, H, backbone), 1.50 (s, 6H, -(CH_3_)_2_), 1.27 (s, 3H, -H), 1.27 (s, H, backbone), 0.87 (s, 9H, -(CH_3_)_2_); GPC (**12**, DMF): 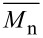 = 54700, 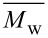 = 182,700, *D* = 3.3; GPC (**13**, DMF): 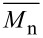 = 68,400, 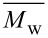 = 218,700, *D* = 3.2.

## Supporting Information

File 1 Additional ITC results, 2D NMR ROESY and hydrodynamic diameters.
