# Supplementary material for: Influence of cyclodextrin on the solubility and the polymerization of (meth)acrylated Triton® X-100
Source: Beilstein J Org Chem. 2012 Dec 13;8:2176–83. doi: 10.3762/bjoc.8.245 (PMC3555431; doi:10.3762/bjoc.8.245)
Supplement: File 1 — Additional ITC results, 2D NMR ROESY and hydrodynamic diameters. [file Beilstein_J_Org_Chem-08-2176-s001.pdf]

Supporting Information

for

Influence of cyclodextrin on the solubility and the polymerization of (meth)acrylated Triton® X-100

Melanie Kemnitz and Helmut Ritter*

Address: Institute of Organic Chemistry and Macromolecular Chemistry II, Heinrich-Heine-University, Universitätsstraße 1, 40255 Düsseldorf (Germany)

Email: Helmut Ritter* - h.ritter@uni-duesseldorf.de

* Corresponding author

Additional ITC results, 2D NMR ROESY and hydrodynamic diameters

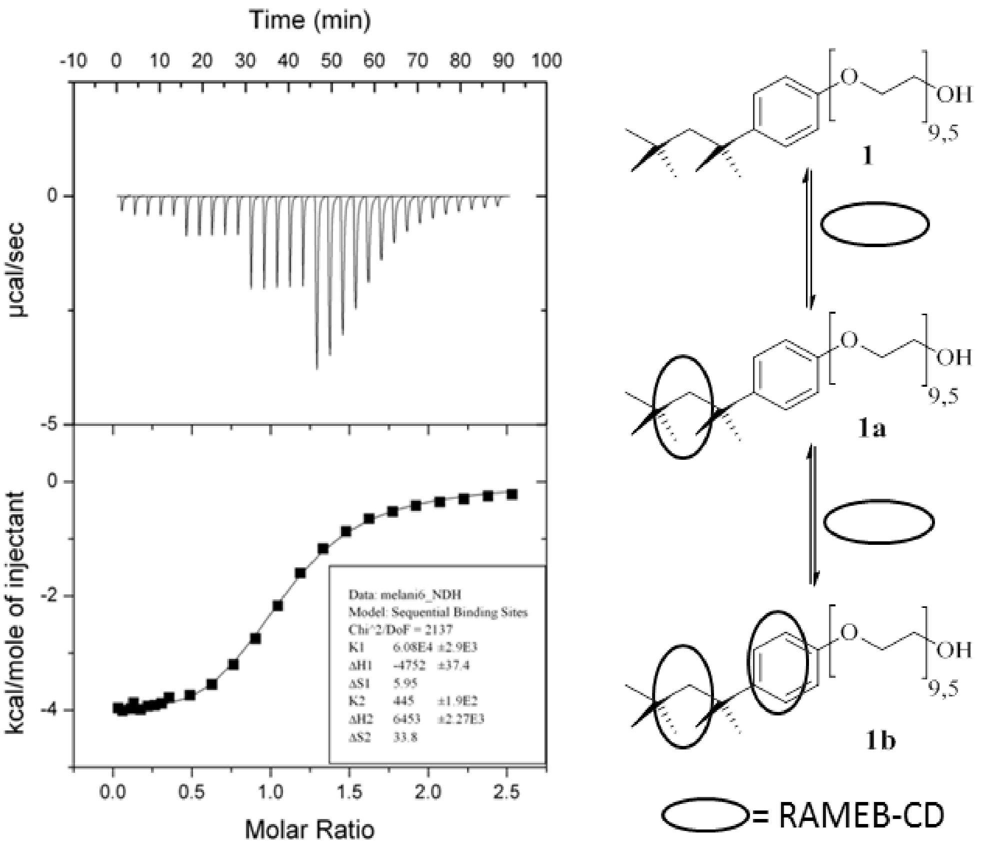

Figure S1: ITC result for a titration of a 2.5 mM solution of RAMEB-CD against a 0.2 mM solution of Triton® X-100 (**1**) in water.

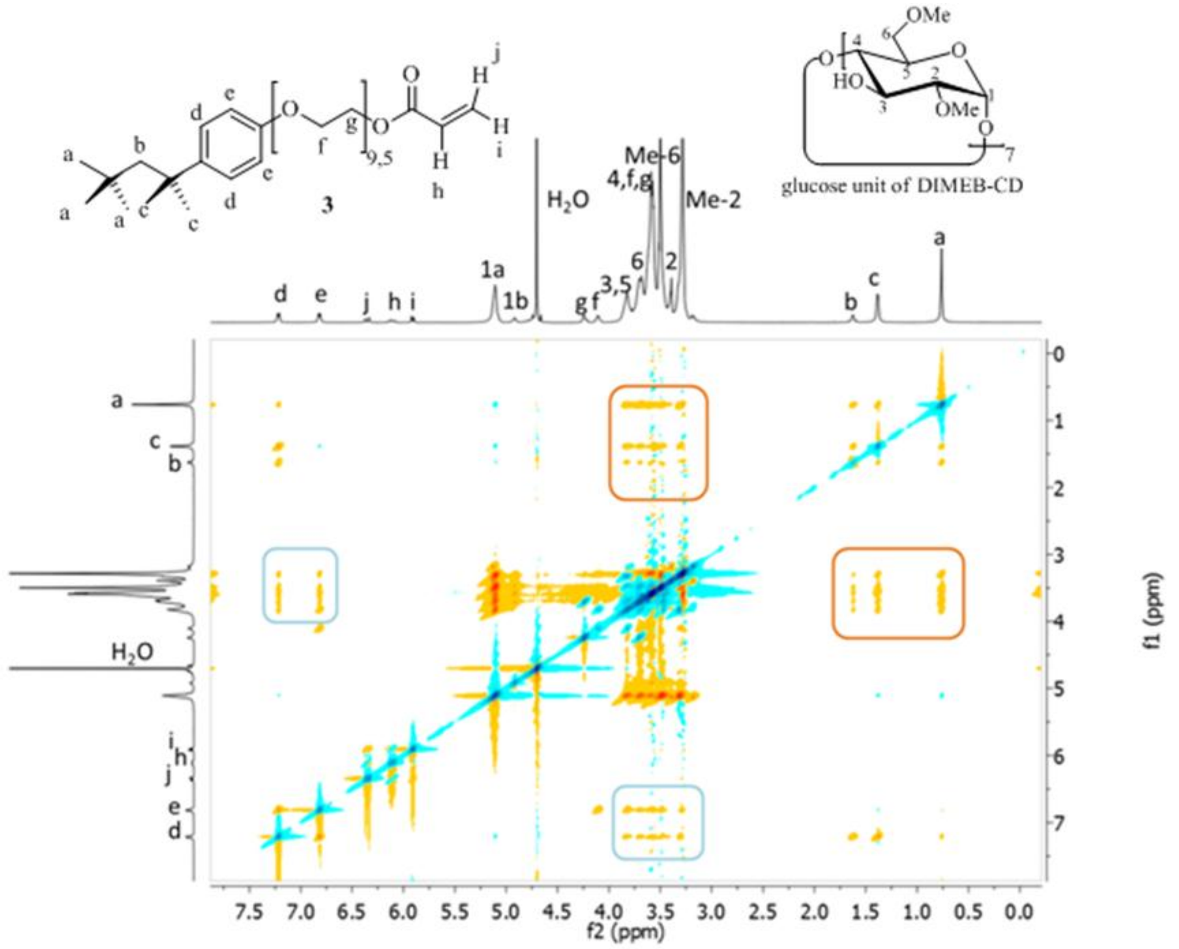

Figure S2: 2D-NMR-ROESY spectrum of the complex between the acrylic monomer derived from Triton® **3** with 2 equiv of DIMEB-CD (300 MHz, D_2O).

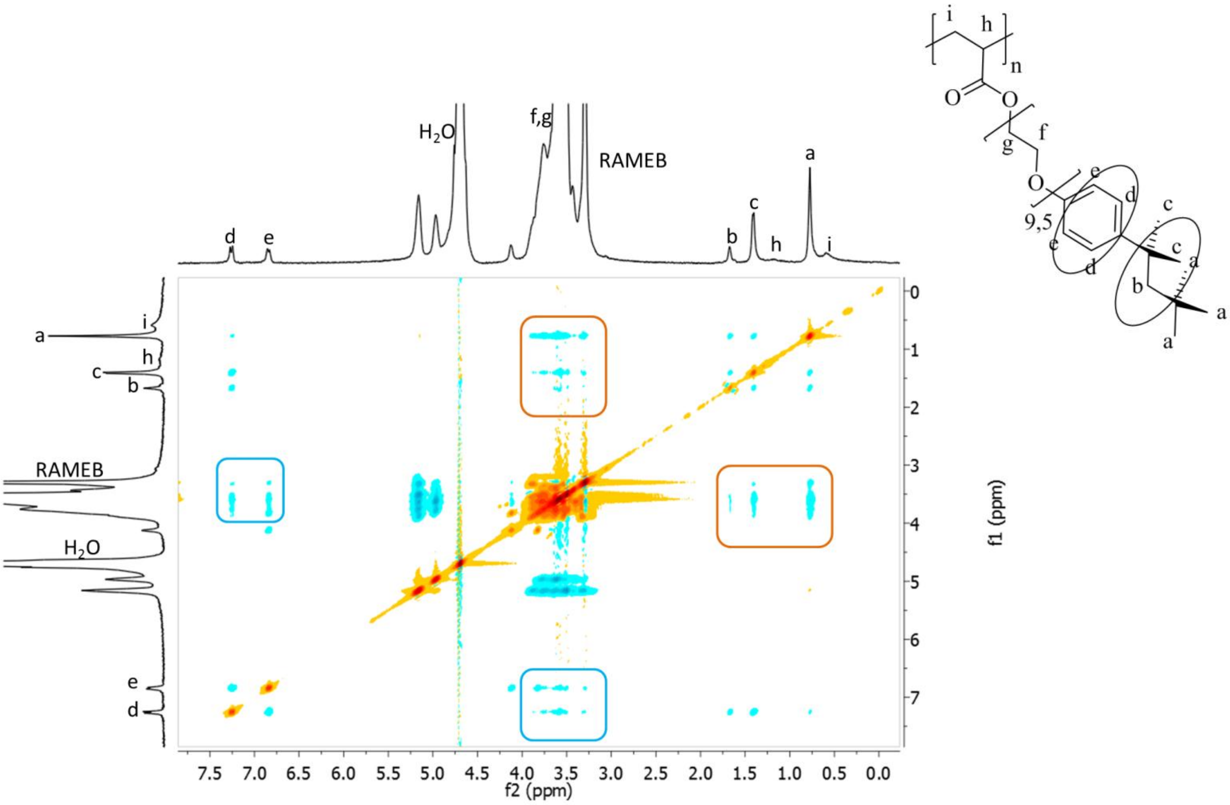

Figure S3: 2D-NMR-ROESY spectrum of the acrylic polymer with two equivalents RAMEB-CD **13** after its polymerization with VA-044 as radical initiator in water (300 MHz, D_2O).

Table S1: The hydrodynamic diameters of the different polymers and their complexes with RAMEB-CD in DMF.

compound	hydrodynamic diameter (nm) in DMF
**8** + 1 equiv of RAMEB-CD[a]	11.4
**10**[b]	11.8
**8** + 2 equiv of RAMEB-CD[a]	18.4
**11**[b]	17.3
**9** + 1 equiv of RAMEB-CD[a]	12.5
**12**[b]	10.2
**9** + 2 equiv of RAMEB-CD[a]	16.3
**13**[b]	15.5

[a]The polymers were homopolymerized in DMF and then complexed with equiv of RAMEB-CD.

[b]First the monomers were complexed with RAMEB-CD and then polymerized in water.
